# CD4^+^ T Cell Fate in Glomerulonephritis: A Tale of Th1, Th17, and Novel Treg Subtypes

**DOI:** 10.1155/2016/5393894

**Published:** 2016-11-15

**Authors:** Christan F. Krebs, Oliver M. Steinmetz

**Affiliations:** III. Medizinische Klinik, Universitätsklinikum Eppendorf, Hamburg, Germany

## Abstract

Multiple studies have identified CD4^+^ T cells as central players of glomerulonephritis (GN). Cells of the Th1 and Th17 responses cause renal tissue damage, while Tregs mediate protection. Recently, a high degree of plasticity among these T cell lineages was proposed. During inflammation, Th17 cells were shown to have the potential of transdifferentiation into Th1, Th2, or alternatively anti-inflammatory Tr1 cells. Currently available data from studies in GN, however, do not indicate relevant Th17 to Th1 or Th2 conversion, leaving the Th17 cell fate enigmatic. Tregs, on the other hand, were speculated to transdifferentiate into Th17 cells. Again, data from GN do not support this concept. Rather, it seems that previously unrecognized subspecialized effector Treg lineages exist. These include Th1 specific Treg1 as well as Th17 directed Treg17 cells. Furthermore, a bifunctional Treg subpopulation was recently identified in GN, which secrets IL-17 and coexpresses Foxp3 together with the Th17 characteristic transcription factor ROR*γ*t. Similarities between these different and highly specialized effector Treg subpopulations with the corresponding T helper effector cell lineages might have resulted in previous misinterpretation as Treg transdifferentiation. In summary, Th17 cells have a relatively stable phenotype during GN, while, in the case of Tregs, currently available data suggest lineage heterogeneity rather than plasticity.

## 1. Introduction

Glomerulonephritides (GNs) comprise a group of complex and heterogeneous disease entities, caused by many different underlying conditions. These include primary forms, for example, IgA nephropathy or membranous GN, as well as secondary forms developing as a consequence of systemic diseases as lupus nephritis and ANCA-associated vasculitides [1]. However, regardless of their etiology, GNs have in common the fact that they are the result of misdirected immune responses. Therefore, in most forms of GN, a pronounced renal glomerular and often also tubulointerstitial inflammatory cell infiltrate is found. Numerous studies from the past have shown that CD4^+^ T cells are crucial mediators of most forms of GN [2].

It has been shown that especially cells of the Th1 and Th17 responses are highly nephritogenic [3–8]. Dysregulated systemic Th1 and Th17 immunity was proven to be central for development of autoimmunity and initiation of GN. In addition, both T helper cell lineages are important mediators of local renal tissue injury as well [9–11]. In contrast to pathogenic Th1 and Th17 responses, regulatory T cells (Tregs) were proven to be potent anti-inflammatory players in GN. Several studies of the past have highlighted their protective effects [7, 12–14].

Given the central roles of T helper effector and T regulatory cells in GN, it is well worthwhile to study their biology and function in detail. Interestingly, increasing evidence suggests that systemic expansion and renal infiltration of different CD4^+^ T cell subtypes follow a concerted time course. Th17 cells were shown to be involved very early in inflammation [15–17]. After rapid renal and systemic expansion, their percentages, however, steadily decrease over time and often decline to reach baseline levels. Cells of Th1 polarization, in contrast, occur later during renal inflammation. They expand at a somewhat slower rate than Th17 cells, but their numbers seem to persist at high levels [16]. Finally, Tregs were shown to steadily expand in a continuous process until a stable equilibrium with their proinflammatory Th1 and Th17 counterparts is established [12, 17].

This defined time course of renal infiltration, which is initially dominated by Th17 cells, followed by Th1 cells and Tregs at later stages, has led to speculations of possible transdifferentiation of Th17 cells into another cell type. One possibility, which has been suggested, is reprogramming of Th17 cells to acquire a Th1 phenotype [18, 19]. Alternatively, Th17 cells could lose their pathogenic properties and might be reprogrammed to become Foxp3^+^ Tregs.

## 2. Concept of CD4^+^ T Cell Plasticity

The change in characteristics of single CD4^+^ T cells, that is, plasticity, has been addressed using different methods over the past decade in human and mouse.* In vitro* approaches as well as transfer experiments using highly purified populations of CD4^+^ T cell subsets have established the view that CD4^+^ T cells can change their polarity under certain conditions [18, 20–26]. To follow the fate of single CD4^+^ T cells, lineage-tracing systems using Cre-recombinase expression under the control of key cytokines or transcription factors and subsequent permanent fluorochrome expression have been established [19, 27–29]. These “fate reporter” mice overcome technical limitations in single cell tracing, which were present in transfer experiments using highly purified or even bulk populations of* in vitro* polarized T cell subsets.

In very elegant studies with IL-17A-Cre fate reporter mice, Hirota et al. have established the concept that encephalitogenic Th17 cells have a high degree of plasticity into the Th1 phenotype in experimental autoimmune encephalomyelitis (EAE), the mouse model for multiple sclerosis [19]. Furthermore, studies in these mice have revealed that, in specialized environments, namely, intestinal Peyer's Patches, Th17 cells potentially develop into T follicular helper cells (Tfh) and drive antigen-specific IgA responses in germinal center B cells [30]. Moreover, regulatory type 1 cells (Tr1), an intriguing T cell subtype with potent immunosuppressive properties, have only recently been recognized as important players in intestinal inflammation. Accumulating evidence suggests that, upon the right stimuli, Th17 cells can transdifferentiate to acquire the ability of IL-10 secretion and become cells with a Tr1 phenotype [31]. A high degree of heterogeneity within certain T cell subsets was also apparent in studies that performed single cell sequencing of Th17 cells from EAE and from* in vitro* culture [32, 33]. Plasticity of human CD4^+^ T cells, on the other hand, can be addressed by using T cell receptors (TCR) as an endogenous barcoding system. Sequencing of TCR revealed a great diversity in the phenotype of cells that presumably descend from a single CD4^+^ T, cell indicating potential transdifferentiation [34, 35]. Studies that focus on plasticity of human CD4^+^ T cells have been reviewed recently in detail by DuPage and Bluestone [36].

In summary, increasing data suggest instability or plasticity, especially, of Th17 cells. However, to complicate things, numerous studies have also postulated a diametrically opposite concept; namely, Th17 cells might derive from transdifferentiation of Foxp3^+^ Tregs [29, 37–40]. The following paragraphs will summarize our current knowledge of CD4^+^ T cell plasticity with a particular focus on glomerulonephritis.

## 3. The Fate of Th17 Cells in Glomerulonephritis 

Given the high nephritogenic potential of Th17 cells [6, 41], their plasticity in renal autoimmune disease is of great clinical interest. Two opposing fates have been proposed: transdifferentiation into Th1 cells [19] or alternatively into anti-inflammatory Tr1 cells [31]. Thus, the question clearly arises, if therapeutic interventions targeting Th17 T cells might be of dual benefit, since these could also hamper development of Th1 responses. On the other hand, blockade of Th17 cell development might also interfere with generation of regulatory T cell subsets and thus impede resolution of tissue injury. However, until now only limited data have been published on the potential plasticity of Th17 cells in glomerulonephritis. In a previous study, we have transferred* in vitro *polarized Th1 and Th17 cells into T cell deficient RAG1 knockout mice and analyzed the pathogenicity of these cell types in a planted-antigen model of GN [42]. Analysis of systemic immune responses revealed that only IFN*γ* but no IL-17 or IL-4 was produced by splenocytes after the transfer of Th1 cells. In contrast, some IFN*γ* was also produced by spleen cells after the transfer of Th17 cells, indicating that some Th17 cells might have adopted a Th1 phenotype. It is, however, important to note that T cell pathogenicity rather than plasticity was the primary focus of this study. As a result, certain restrictions limit the interpretation of the results. In particular, the* in vitro* polarized Th17 cells contained a relevant fraction of IFN*γ* producing Th1 cells even before transfer, which clearly limits analysis. Furthermore, only systemic but not organ specific T cell responses in the kidney were addressed. In summary, this study indicates stability of splenic Th1 cells, without significant Th1 to Th17 or Th2 plasticity but suggests some degree of Th17 cell transdifferentiation into cells of the Th1 type during GN. More recent data, however, do no support this latter concept. Tulone et al. traced the fate of* in vitro* Th17 polarized cells in another planted-antigen model of GN [43]. These authors evaluated cytokine expression of splenic and renal T cells after transfer and found somewhat lower Th17 cell frequencies than expected. Importantly, however, they did not detect sizeable fractions of Th1 cells. These findings therefore indicate partial loss of the Th17 effector phenotype but do not support significant Th17 to Th1 transdifferentiation.

In a recent study, we have addressed the plasticity of renal Th17 cells in more detail [44]. After transfer of highly purified* in vitro* polarized Th17 cells from fluorescence reporter mice [45] and subsequent induction of crescentic glomerulonephritis, reanalysis of T cells from the kidney displayed a relatively high degree of stability. Moreover, using IL-17A fate reporter mice [19], these findings were confirmed in immunocompetent mice in two models of experimental glomerulonephritis. Importantly, in these studies, no relevant transdifferentiation into Th1 or Th2 cells was detected among ex-Th17 cells [44], leaving their fate unknown. In this context, it is important to note that Th17 cells have been reported to have a high rate of instability and conversion into Th1 cells in nonrenal models of autoimmune diseases [19]. This indicates that the kidney provides a unique environment that supports the stability of Th17 cells.

Since Th17 cells are pathogenic in crescentic glomerulonephritis [6, 41], we aimed at actively interfering with their stability. We thus treated mice with a monoclonal anti-CD3 antibody [46], which resulted in induction of a tolerogenic phenotype, characterized by IL-10 coexpression, in otherwise stable renal Th17 cells. The great therapeutic potential of this finding for the treatment of renal autoimmune diseases clearly warrants further exploration.

**Figure 1:**
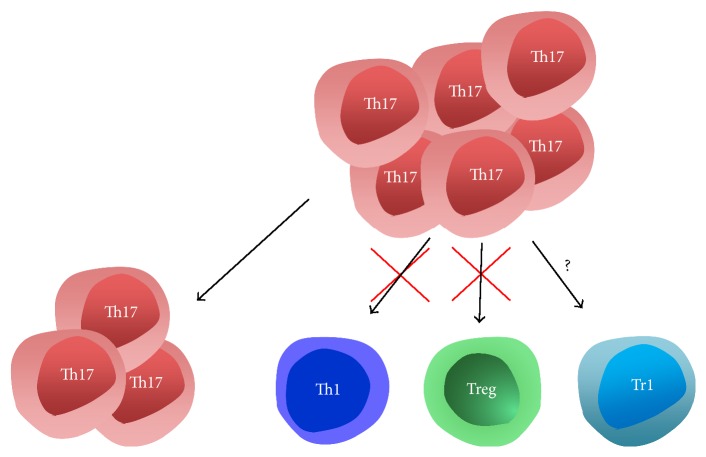
The fate of Th17 cells during glomerulonephritis remains unknown. While some Th17 cells conserve their phenotype, a relevant fraction of Th17 cells seems to change their fate. Currently available data, however, do not support predominant conversion into either Th1 cells or Foxp3^+^ Tregs. An intriguing possibility that remains might be adoption of a Tr1 phenotype.

Since renal Th17 cells do not seem to acquire either Th1 or Th2 phenotypes, an alternative scenario would be Th17 transdifferentiation into anti-inflammatory Foxp3^+^ Tregs. However, available data from renal disease do not support this notion either. In a recent study, we have transferred Treg depleted spleen cells into RAG1-deficient mice and induced a model of accelerated crescentic glomerulonephritis [7]. Interestingly, flow cytometry of splenocytes revealed persistent absence of FoxP3^+^ cells at the end of the experiment. This finding indicates that there is no transdifferentiation of non-Treg T cell subsets, including Th17 cells, into FoxP3^+^ Tregs in this model. Furthermore, in another study, we transferred highly purified Th17 cells into lymphocyte intact mice and traced their fate during GN, using a congenic marker system. Interestingly, most of the transferred Th17 cells had lost their ROR*γ*t expression. Importantly, though, none of these ex-Th17 cells had upregulated Foxp3 in kidneys or spleens, which excludes transdifferentiation into Tregs [17]. In summary, increasing evidence supports some degree of Th17 instability during GN, which, however, does not lead to generation of Th1 cells or Tregs. On possible scenario would thus be that Th17 cells adopt a Tr1 phenotype as has recently been suggested [31]. An overview is given in Figure 1. Further studies are, however, clearly needed.

## 4. Treg Stability: A Matter of Debate

Another much discussed aspect of CD4^+^ T cell plasticity is the hypothesis that Foxp3^+^ Tregs might possess the capacity to transdifferentiate into T effector cells. This notion was supported by early experiments, in which Tregs were highly purified from Foxp3 fluorescence reporter mouse strains and transferred into different T cell deficient recipients [37]. Surprisingly, around half of the transferred Tregs had lost Foxp3 expression, as well as other Treg hallmark molecules as CD25 and CTLA-4. Assessment of their function revealed loss of suppressive capacity, and even more suggestive of transdifferentiation, these ex-Tregs had started to produce inflammatory cytokines as IL-17 and IFN*γ*. Further rounds of experiments, however, made clear that this observed high degree of instability was overestimated due to the lymphopenic environment in the recipient mice. When Tregs were cotransferred with T helper effector cells or transferred into lymphocyte intact recipients, only few Tregs lost their phenotype [37, 40, 47]. This new and refined view on Treg plasticity was confirmed by later elegant studies using Foxp3 fate reporter mice. These studies from different groups consistently reported a high degree of Treg stability with, however, minor populations of between 5 and 10% showing loss of Foxp3 [29, 48–50]. The fate of these ex-Foxp3 cells is currently unsolved and remains a highly discussed topic [51]. Another observation adds further complexity to Treg biology. While most Tregs seem to stably maintain Foxp3 expression, substantial fractions were found to coexpress master transcription factors and key cytokines of T helper effector cell lineages [52–54]. Four possible scenarios regarding these T cells with a hybrid phenotype are currently under debate. Firstly, effector T helper cells could transiently coexpress Foxp3 during early stages of activation [49, 55, 56]. Secondly, they could be Tregs transdifferentiating into T helper effector cells. Alternatively, Tregs might be upregulating classical T helper effector cell transcription factors to gain certain functional characteristics and, last but not least, they could be independent and previously unrecognized T cell lineages. To date, not many published data support the first and second concepts. In contrast, an increasing body of evidence, including data from human and experimental glomerulonephritis, points towards the third hypothesis [57–60]. In analogy to lineage diversity among T helper effector cells, different corresponding Treg subtypes might exist. Lineage specificity of these Th1-, Th2-, or Th17-type Tregs seems to be achieved by sharing some transcription factors with their respective T helper effector counterpart [14, 61–63]. Th1 specific Treg1 cells coexpress the Th1 master transcription factor T-bet [53, 60] and Treg17 cells, targeting Th17 responses, rely on coactivation of Stat3 [52, 57, 58]. Finally, very recent data suggest existence of a third and independent T cell lineage, different from T helper effector cells and conventional Tregs, which coexpresses the unusual combination of the Treg master transcription factor Foxp3 with the Th17 characteristic ROR*γ*t [17, 54, 64, 65]. The following paragraphs will summarize available data on Treg diversification and their subphenotypes and stability in GN.

## 5. Lineage Specific Tregs: One Size Does Not Fit All

Landmark studies by Chaudhry et al.'s group have suggested that Tregs can coactivate the Th17 characteristic transcription factor Stat3 [52]. This prompted the question whether Stat3 and Foxp3 double positive cells might be Tregs transdifferentiating into Th17 cells or even bifunctional Treg/Th17 hybrids. If either of these concepts was true, preventing Stat3 activation in Tregs should result in reduced Th17 immunity. However, this was not the case. In contrast, specific deletion of Stat3 in Tregs surprisingly resulted in spontaneous exacerbation of Th17 immunity and development of fatal colitis [52]. This indicated control of Th17 responses, specifically by a subset of Tregs, which coexpresses Stat3. Furthermore, this finding supported previously unrecognized Treg heterogeneity, rather than instability or transdifferentiation. Since nothing was known about the role of these Th17 specific Stat3 dependent Treg17 cells in inflammatory diseases, our group studied their function in experimental GN. In line with the data by Chaudhry et al., we found that loss of Stat3 in Tregs significantly enhanced type-17 immunity and aggravated renal disease in models of acute crescentic GN [52] and chronically developing systemic lupus erythematosus [58]. Importantly, the enhanced levels of IL-17 were not a result of cytokine production by Tregs but rather by Foxp3 negative Th17 cells. Furthermore, Treg numbers and percentages in blood and spleens were not reduced, indicating preserved Treg stability. Interestingly, however, we found significantly impaired Treg accumulation in the inflamed kidneys. As an underlying mechanism, we could identify lack of the chemokine receptor CCR6 on Stat3 deficient Tregs. This trafficking receptor is characteristically found on Th17 cells and mediates their infiltration into inflamed kidneys [11]. Stat3 mediated expression of CCR6 on Tregs therefore enables their trafficking into areas of Th17 inflammation and facilitates close cell contacts which optimizes direct immunosuppression. Importantly, this mechanism seems to be conserved across species. Studies in humans confirmed close colocalization of CCR6 positive Tregs with CCR6 positive Th17 cells in kidneys of patients with ANCA-associated GN. Furthermore, analyses of blood from patients with Hyper-IgE Syndrome (HIES), caused by dominant negative Stat3 mutations, showed normal Treg percentages but significant reduction of Treg expressed CCR6 [57]. Collectively, these observations indicate that Stat3 activation, both in mice and in humans, does not reflect Treg reprogramming or instability but rather specialization for counterregulating Th17 immunity.

**Figure 2:**
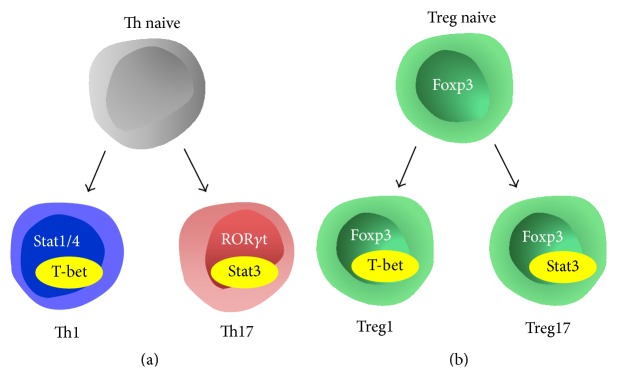
(a) Naive T helper cells can acquire a Th1 phenotype by activating the transcription factors Stat1 and Stat4 together with T-bet. A Th17 phenotype requires activation of Stat3 in combination with ROR*γ*t. (b) In analogy to T helper cell fate, a naive Foxp3^+^ Treg can activate T-bet under inflammatory conditions. This process does not result in Treg/Th1 transdifferentiation but rather induces a Treg1 phenotype, which optimizes Treg properties for control of Th1 responses. Likewise coactivation of Stat3 with Foxp3 generates Treg17 cells, which are tailor made to suppress Th17 immunity.

Similar to activation of the Th17 characteristic transcription factor Stat3, pioneering studies by Koch et al. have noted coexpression of Foxp3 with the Th1 master inducer T-bet [53]. Again, the question arose whether this might represent transdifferentiation of Tregs into Th1 cells. This hypothesis was supported by the observation that T-bet^+^ Tregs express large amounts of the Th1-type cytokine IFN*γ*. However, the opposite was the case. Elaborate transfer studies, using Tregs from T-bet pan knockout mice, showed that T-bet confers Tregs with the capacity to effectively downregulate type-1 immunity. Absence of Treg1 cells resulted in overshooting Th1 responses, underlining their regulatory rather than proinflammatory function [53]. In analogy to the shared expression of CCR6 by Stat3 positive Th17 and Treg17 cells, T-bet positive Th1 and Treg1 cells were shown to share the chemokine receptor CXCR3, which facilitates their colocalization [53]. Importantly, Treg1 cells arose from T-bet negative uncommitted Tregs, which activated T-bet during inflammation and not from transient promiscuous upregulation of Foxp3 in Th1 cells. Taken together, T-bet expressing Tregs do not seem to be transdifferentiating intermediates on their way to a Th1 phenotype but rather constitute a Th1 specialized suppressor population. Not much is known regarding Treg1 cells in renal disease. Our group therefore addressed this question and induced experimental crescentic GN in mice with a Treg selective defect in T-bet activation. If T-bet expression in Tregs resulted in transdifferentiation towards Th1 cells, Treg numbers in these mice should increase, while Th1 responses should be diminished. However, Treg homeostasis was not impaired and systemic Treg frequencies were normal. Furthermore, instead of reduced numbers of Th1 cells, mice with a Treg specific defect of T-bet activation developed significantly overshooting Th1 immunity and showed worsened glomerular disease [60]. These observations clearly refute the concept of Treg/Th1 transdifferentiation and support the view of T-bet^+^ Tregs as effector Treg population, specialized for the control of pathogenic Th1-type inflammation. Taken together the available data from studies of GN suggest Treg lineage heterogeneity and specialization during organ inflammation rather than instability or plasticity. An illustration of this concept is shown in Figure 2.

## 6. ROR***γ***t^+^ Tregs: The New Cells on the Block 

The observation that Tregs can coactivate certain transcription factors of T helper effector cell lineages has led to the discovery of cells, expressing Foxp3 together with the Th17 master transcription factor ROR*γ*t [54]. These ROR*γ*t^+^ Tregs were found to be present not only in mice but also in healthy humans as well as in many inflammatory conditions [66–68].

**Figure 3:**
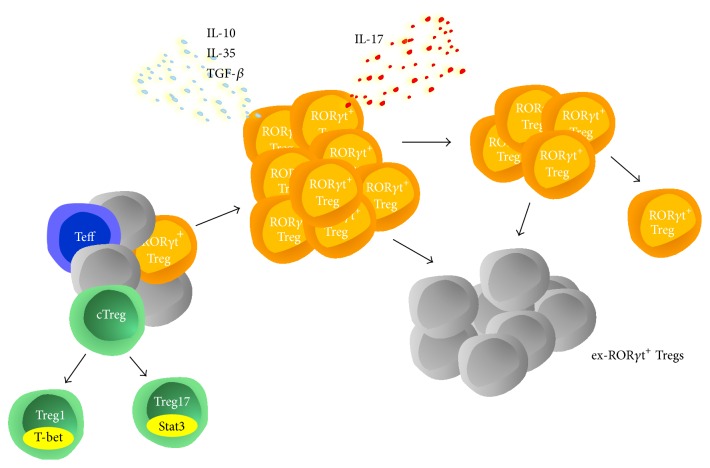
The immune system gives rise to different CD4^+^ T cell lineages. Among these are Th effector cells (Teff), which can differentiate into Th1 and Th17 cells during inflammation. Furthermore, there are conventional ROR*γ*t^−^Foxp3^+^ Tregs (cTreg), which have the potential to develop into specialized Treg1 and Treg17 cells. Finally, a third and hitherto unrecognized independent Treg cell lineage exists, which is characterized by simultaneous expression of ROR*γ*t and Foxp3. During inflammation, for example, glomerulonephritis, they rapidly expand by proliferation and start to produce great amounts of pro- and anti-inflammatory cytokines. Subsequently, their population retracts by downregulating both transcription factors, ROR*γ*t and Foxp3, to become ex-ROR*γ*t^+^ Tregs. The functional properties of these ex-ROR*γ*t^+^ Tregs remain elusive to date.

Several authors had suggested before that some Treg subpopulations might transdifferentiate into Th17 cells, making cells that coexpress Foxp3 with ROR*γ*t likely candidates [29, 37–40, 48]. Our group therefore decided to address this open question and studied origin, function, and plasticity of ROR*γ*t^+^Foxp3^+^ T cells. During different models of GN, we found that ROR*γ*t^+^ Tregs massively expanded systemically and also in the nephritic kidneys, early during the course of inflammation [17]. Interestingly, analysis of the ROR*γ*t^+^ Tregs cytokine profile revealed production of both, Treg characteristic cytokines TGF-*β*, IL-35/EBI-3, and IL-10 and the Th17 hallmark cytokine IL-17A. Since this observation indeed suggested that ROR*γ*t^+^ Tregs are cells undergoing transdifferentiation, either from Th17 into Tregs or the other way around, we performed transfer studies. Transfer of highly purified ROR*γ*t^+^Foxp3^−^ Th17 cells or ROR*γ*t^−^ Foxp3^+^ conventional Tregs into congenic lymphocyte intact recipients revealed that none of the single transcription factor positive cells upregulated the other transcription factor during the course of experimental GN. This observation surprisingly indicated that ROR*γ*t^+^ Tregs did not derive from Treg/Th17 transdifferentiation but rather represent an independent cell lineage. However, a second hypothesis, explaining the origin of ROR*γ*t^+^ Tregs, might be transient and promiscuous upregulation of Foxp3 during early activation of Th17 cells [49, 55, 56]. In order to test this possibility, we generated fate reporter mice, in which cells are permanently marked, once they have upregulated Foxp3 at any state of development. Antigen challenge of these mice, however, showed that virtually all cells, which had activated Foxp3, indeed remained Foxp3 positive during the following activation period of 7 days. We could thus exclude transient Foxp3 upregulation in ROR*γ*t^+^ Th17 cells, as event leading to generation of ROR*γ*t^+^Foxp3^+^ Tregs [17]. A third possible scenario, underlying the origin of ROR*γ*t^+^ Tregs, however, remained. Since ROR*γ*t is a downstream target of Stat3, we wanted to evaluate whether ROR*γ*t^+^ Tregs might possibly resemble Treg17 cells, which we had previously shown to be induced by activation of Stat3 [57, 58]. Analysis of mice with a Treg specific deficiency of Stat3 activation, however, showed unaltered ROR*γ*t expression in Tregs in both spleens and nephritic kidneys, excluding this possibility and further underlining the independent nature of ROR*γ*t^+^ Tregs. Next, we decided to generate mice with selectively impaired ROR*γ*t activation in Foxp3^+^ Tregs. If ROR*γ*t^+^ Tregs were Treg to Th17 transdifferentiating cells, this transdifferentiation would be impaired in these mice and they should progressively accumulate Tregs, while Th17 cell numbers should decline over time. In case that bi-Tregs resembled Treg17 cells, Th17 responses should be overshooting in mice with deficient ROR*γ*t activation in Tregs. Analyses at different time points in different models of GN, however, revealed unchanged Treg and Th17 cell homeostasis. Again, this observation indicated an independent nature of ROR*γ*t^+^ Tregs, with no signs of Th17 transdifferentiation or Treg17 specialization [17]. Finally, we wanted to study the direct fate of ROR*γ*t^+^ Tregs. For this purpose, they were highly purified by flow cytometric sorting and subsequently transferred into congenic recipient mice. 10 days after induction of GN, the transferred cells were reanalyzed in spleens and nephritic kidneys. Our results showed that the transferred ROR*γ*t^+^ Tregs had massively expanded in both organs during inflammation. Much to our surprise, the vast majority had lost both transcription factors, ROR*γ*t and Foxp3, to become double negative (ex-ROR*γ*t^+^ Tregs). Importantly, however, we did not observe relevant transdifferentiation of ROR*γ*t^+^ Tregs into either ROR*γ*t^+^Foxp3^−^ Th17 cells or conventional ROR*γ*t^−^Foxp3^+^ Tregs, leaving the fate of ex-ROR*γ*t^+^ Tregs unknown [17]. Taken together, our studies thus support the following concepts: (1) ROR*γ*t^+^ Tregs are not Tregs transdifferentiating into Th17 cells, (2) ROR*γ*t^+^ Tregs are not Th17 cells on their way to become Tregs, (3) ROR*γ*t^+^ Tregs are not Th17 cells transiently expressing Foxp3 during activation, and (4) ROR*γ*t^+^ Tregs do not resemble Th17 specific Treg17 cells. Rather, they represent an independent effector Treg lineage, which rapidly expands during inflammation and subsequently retracts by as to yet unknown mechanisms. Based on currently available data, we thus propose the concept outlined in Figure 3.

The independent nature of ROR*γ*t^+^ Tregs has recently been confirmed by three highly ranked studies [64, 65, 69]. After transfer into lymphopenic Rag mice, ROR*γ*t^+^ Tregs showed a high degree of stability with no relevant transdifferentiation into Th17 cells or conventional Tregs [69].

With respect to the function of ROR*γ*t^+^ Tregs, concepts are, however, still evolving. Our studies have shown that exogenously transferred ROR*γ*t^+^ Tregs were highly protective in a model of acute GN. However, endogenous ROR*γ*t^+^ Tregs had additional proinflammatory functions. Selective inhibition of ROR*γ*t activation in Tregs resulted in complete abrogation of their IL-17 production and ameliorated renal tissue damage in acute GN [17]. In addition, the recent landmark report by Ohnmacht et al. surprisingly showed that ROR*γ*t expression in Tregs is crucial for suppression of Th2 immunity [64]. Our own previous work in a model of acute crescentic GN [7], and unpublished data using the pristane model of murine SLE, strongly supports this observation. ROR*γ*t^+^ Tregs might thus be potent regulators of Th2 responses and could be important to protect from allergies [64]. In summary, Foxp3 and ROR*γ*t coexpressing Tregs are not Treg/Th17 transdifferentiating or Th17 specialized Treg17 cells but represent a unique, stable, and independent T cell lineage with both regulatory and proinflammatory functions.

## 7. Conclusions 

A thorough understanding of CD4^+^ T cell plasticity is of high clinical relevance in the light of newly emerging T helper cell directed therapies. While Th1 cells display a relatively high degree of stability in GN, Th17 cells may have different fates. In several experimental models of nonrenal inflammatory disease, most Th17 cells transdifferentiate into a Th1 phenotype. In GN, however, a significant proportion of Th17 cells seems to be stable and maintains the differentiation status. Another fraction, in contrast, appears to undergo functional changes and loses Th17 characteristics. Nevertheless, currently available data do not support Th17 conversion into Th1, Th2, or Treg cells during the course of GN, leaving the nature of ex-Th17 cells unknown. Interestingly, however, Th17 cells can be pushed into a tolerogenic phenotype by anti-CD3 treatment, which represents a promising therapeutic strategy. In the case of Tregs, data from GN underline a very high degree of stability and show no hint for transdifferentiation. Rather, a new paradigm is emerging, suggesting activation and diversification of naive Tregs into different effector Treg lineages as ROR*γ*t^+^ Tregs, T-bet^+^ Treg1, and Stat3^+^ Treg17 cells.
